# Emergency department outpatient treatment of alcohol-intoxicated bicyclists increases the cost of medical care in Japan

**DOI:** 10.1371/journal.pone.0174408

**Published:** 2017-03-22

**Authors:** Yosuke Homma, Sunao Yamauchi, Michiko Mizobe, Yoshiyuki Nakashima, Jin Takahashi, Hiraku Funakoshi, Kevin Y. Urayama, Sachiko Ohde, Osamu Takahashi, Takashi Shiga

**Affiliations:** 1 Department of Emergency Medicine and Critical Care, Tokyo Bay Urayasu Ichikawa Medical Center, Chiba, Japan; 2 Department of Emergency Medicine, University of Iowa Hospitals and Clinics, Iowa City, IA, United States of America; 3 Center for Clinical Epidemiology, St. Luke’s International University, Tokyo, Japan; Beihang University, CHINA

## Abstract

Riding a bicycle under the influence of alcohol is illegal in Japan. Nevertheless, intoxicated bicyclists are frequently treated at hospital emergency departments for bicycle-related injuries. This patient population usually requires more hospital resources, even for relatively minor injuries. Therefore, we hypothesized that bicycle-related crashes involving bicyclists under the influence of alcohol cost more to treat than those that do not involve alcohol intoxication. The aim of the present study was to examine the costs associated with bicycle-related minor injuries and alcohol intoxication of the bicyclist. The study was conducted at the Tokyo Bay Urayasu Ichikawa Medical Center Emergency Department, Japan. All minor bicycle crashes involving 217 individuals aged ≥20 years treated from September 1, 2012 to August 31, 2013 were included in the analysis of data obtained from medical records. Variables included alcohol intoxication, sex, age, collision with a motor vehicle, Glasgow Coma Scale, injury severity score (ISS), laboratory tests, treatment of wounds, number of X-ray images, number of computed tomography scans, and medical costs. Multiple linear regression analysis was performed to evaluate the association between alcohol intoxication and medical costs. Seventy (32%) patients consumed alcohol, and the median medical cost was 253 USD (interquartile range [IQR], 164–330). Multivariable analysis showed that alcohol intoxication was independently associated with higher medical costs (*p* = 0.030, adjusted R-square value = 0.55). These findings support our hypothesis and should encourage authorities to implement comprehensive measures to prohibit bicycling under the influence of alcohol to prevent injuries and to reduce medical costs.

## Introduction

Approximately 70 million people in Japan own bicycles[1], and the use of bicycles for commuting, sports, and leisure is encouraged worldwide[2]. However, in Japan, bicycle-related crashes occur with a frequency of approximately 100,000 per year[3], and in the United States, approximately 480,000 people are treated annually at emergency departments (ED) for bicycle-related injuries[4]. Despite the laws which prohibit riding a bicycle under the influence of alcohol[5], bicycle-related injuries suffered by intoxicated riders are frequent. Moreover, bicycle-related crashes occur more frequently when bicyclists are under the influence of alcohol[6, 7].

Alcohol intoxication is associated with higher medical costs when patients are hospitalized or discharged by bicycle-related injuries[8, 9]. However, the relationship between alcohol intoxication and medical costs focused on outpatient minor injuries has not been extensively evaluated. Therefore, the aim of this study was to assess the association between alcohol intoxication and medical costs among patients affected with minor bicycle-related injuries.

## Methods

### Study design and subjects

This study was approved by the ethics committee of the Tokyo Bay Urayasu Ichikawa Medical Center (approval no: 13) and was implemented in accordance with the provisions of the Declaration of Helsinki. Patient information was anonymized and de-identified prior to analysis, and the need for patient consent was waived. This study was institutional review board-approved and patient consent was exempted because of the retrospective nature.

We conducted a prospective cohort study at Tokyo Bay Urayasu Ichikawa Medical Center, Japan, a large urban general hospital that accepts approximately 8,000 patients annually arriving by ambulance. In Japan, alcohol drinking is legally permitted from the age of 20 years. Thus, the present study included bicyclists aged ≥20 years who were seen at the hospital by ambulance between September 1, 2012 and August 31, 2013 for injuries caused by bicycle crashes. Only patients who were transported to the ED in an ambulance were included because of the difference in calculating medical costs between them and those who arrived using other means. Patients who were hospitalized or died were excluded due to our focus on “minor injuries” defined as care through the outpatient mechanism; there is a difference in medical costs required to care for inpatients compared with outpatients. To calculate costs, the observation period of this study started on the day of arrival of the patient to the ED.

### Data collection and measurements

Data were obtained from hospital medical records. By the Japanese road traffic act, riding a bicycle under the influence of alcohol is judged based on whether it affects ability to safely operate the bicycle, and not strictly by blood alcohol concentration[5]. Thus, in this study, we used the same approach where alcohol intoxication was assessed through a physical examination conducted by a physician or emergency medical service staff. This included observing for the odor of alcohol on breath, slurred speech, unsteady gait, impaired gross motor control, decreased alertness, euphoric mood, and/or flushed appearance; assessment was made as potential symptoms of alcohol intoxication and patients with specific medical or mental health conditions that may have overlapping symptoms were not included. Variables included alcohol intoxication, sex, age, type of accident, Glasgow Coma Scale (GCS), injury severity score (ISS)[10], laboratory tests, treatment of wounds, number of X-rays, number of computed tomography (CT) scans, and medical costs (1.00 USD = 124.00 JPY as of August 04, 2015). Type of accident was classified into three categories: collision with a motor vehicle, collision with a bicycle, and collision with still objects. Data on GCS and ISS were collected as an indicator of patient injury severity. GCS was classified into two groups as follows; GCS of 15 as alert wakefulness condition, GCS less than 15 as not alert wakefulness condition. Data on laboratory tests, protocol for treating wounds, number of X-rays, and number of CT scans were collected because of their relatively high contribution to medical costs.

We focused on medical costs provided by the ED, which were calculated as national health insurance (NHI) points. These costs were paid according to a fee-for-service schedule and included physical examinations, laboratory tests, imaging tests, and medical treatment. Costs were calculated only once when the patients were admitted to the ED for the first time of the crash. We obtained the data on medical costs from the patients’ hospital accounts.

### Statistical analysis

Patients’ characteristics were evaluated according to alcohol intoxication classification using Pearson’s chi-square test for categorical variables and the Mann-Whitney U-test for continuous variables, as appropriate. Medical cost values were not transformed for normalization, and the Mann-Whitney U or Kruskal-Wallis test was used to compare median costs across categorical variables. Continuous variables were categorized (based on median value) to evaluate differences in medical costs.

Multiple linear regression analysis was performed to investigate the association between alcohol intoxication and medical costs, while adjusting for potential confounding factors. We calculated adjusted R-squared values to assess the correlation between the actual cost and cost calculated according to the regression coefficients. All statistical analyses were performed using IBM Statistical Package for the Social Sciences version 22.0 (IBM, Corp, Armonk, NY, USA). A *p* value of less than 0.05 was considered statistically significant.

## Results

**Fig 1:**
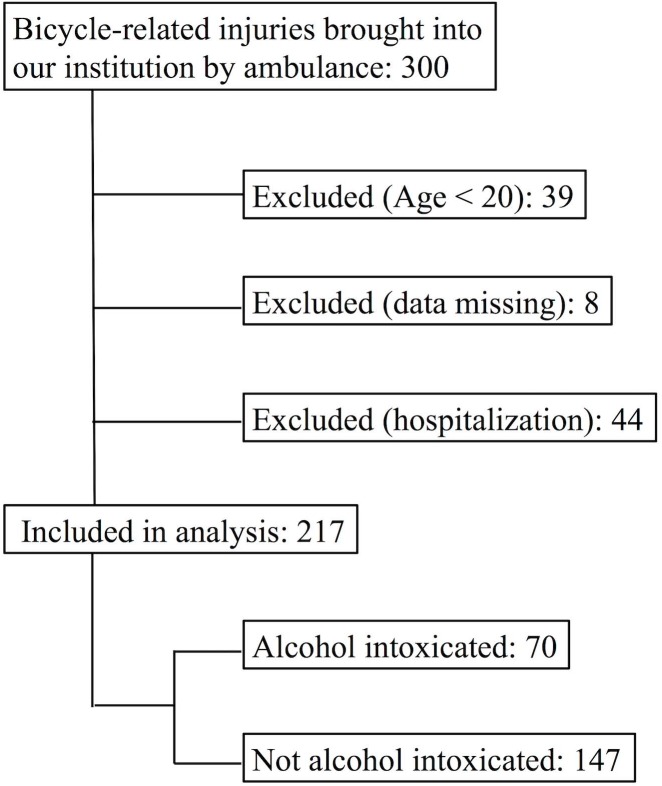
Inclusion criteria of this study.

During the study period, 217 of 300 patients treated at our institution for bicycle-related injuries met the inclusion criteria (Fig 1).

The types of accident included collisions with a motor vehicle (42%), a bicycle (15%), and still objects (43%). The median medical cost was 253 USD (interquartile range [IQR], 164–330 USD) and included the following categories and percentage of cost in decreasing order: imaging tests (55.5%), laboratory tests (9.5%), treatments (8.4%), and others. Over 65% of patients received CT scans (*n* = 145) and X-rays (*n* = 143); a smaller proportion received laboratory tests (*n* = 58, 26.7%), and treatment for wounds (*n* = 79, 36.4%). The regions of CT scan include head (*n* = 131, 90.3%), cervical spine (*n* = 108, 74.5%), face (*n* = 16, 11.0%), chest (*n* = 15, 10.3%), abdomen (*n* = 10, 6.9%), and extremities (*n* = 2, 1.4%).

Baseline characteristics of patients are shown in Table 1. Seventy (32%) patients were intoxicated, and there was no statistically significant difference in age between the alcohol intoxicated group (median age, 53 years [IQR, 35–64]) and not intoxicated group (median age, 45 years [IQR, 34–62]). Bivariate analysis showed sex (*p* < 0.001), type of accident (*p* < 0.001), GCS of 15 (*p* < 0.001), laboratory tests (*p* = 0.009), treatment of wounds (*p* < 0.001), number of X-rays (*p* = 0.015), number of CT scans (*p* < 0.001), head CT scan (*p* < 0.001), cervical spine CT scan (*p* < 0.001), and face CT scan (*p* = 0.049) to be significantly associated with whether or not alcohol intoxicated. The median medical costs were 308 USD (IQR, 232–385) and 225 USD (IQR, 115–296) for injuries involving or not involving alcohol intoxication, respectively, and the difference was statistically significant (*p* < 0.001).

**Table 1:** Bivariate analysis of characteristics associated with alcohol intoxication among bicyclists visiting the ED for minor injuries.

		Not alcohol intoxicated	Alcohol intoxicated	*p*-value*
**n**		147	70	
**Male sex (%)**		72 (49.0)	53 (75.7)	< 0.001
Age, median (IQR**), **years**		45 (34–62)	53 (35–64)	0.141
**Type of accident (%)**				
**Collision with a motor vehicle**		79 (53.7)	12 (17.1)	< 0.001
**Collision with a bicycle**		26 (17.7)	7 (10)	
**Collision with still objects**		42 (28.6)	51 (72.9)	
GCS**) of 15 (%)		142 (96.6)	50 (71.4)	< 0.001
ISS**) score, median (IQR**)		1 (1–2)	1 (1–2)	0.894
**Laboratory tests (%)**		31 (21.1)	27 (38.6)	0.009
**Treatment of wounds (%)**		42 (28.6)	37 (52.9)	< 0.001
Number of X-rays, median (IQR**		1 (0–2)	1 (0–2)	0.015
Number of CT scans, median (IQR**))		1 (0–2)	2 (1–2)	< 0.001
	**Head (%)**	72 (49.0)	59 (84.3)	< 0.001
	**Cervical spine (%)**	58 (39.5)	50 (71.4)	< 0.001
	**Face (%)**	7 (4.8)	9 (12.9)	0.049
	**Chest (%)**	12 (8.2)	3 (4.3)	0.396
	**Abdomen (%)**	7 (4.8)	3 (4.3)	1.000
	**Extremities (%)**	2 (1.4)	0 (0)	1.000
Medical costs, median (IQR**), USD		225 (115–296)	308 (232–385)	< 0.001

*Analyzed using the chi-squared and the Mann-Whitney U-tests.

**GCS, Glasgow Coma Scale; ISS, injury severity score; IQR, interquartile range.

Table 2 shows the association between each variable and medical cost. Male sex (*p* = 0.001), GCS less than 15 (*p* < 0.001), higher ISS score (*p* = 0.007), having laboratory tests (*p* < 0.001), receiving treatment for wounds (*p* < 0.001), higher number of CT scans (*p* < 0.001), and higher number of X-rays (*p* = 0.005) were significantly associated with higher medical costs.

**Table 2:** Bivariate analysis of factors associated with medical costs among bicyclists visiting the ED for minor injuries.

		n (%)	Median (IQR**)	*p*-value*
**Sex**				
	**Male**	125 (57.6)	263 (203–360)	0.001
	**Female**	92 (42.4)	224 (107–287)	
**Age (range)**				
	**20–47 years**	112 (51.6)	245 (133–332)	0.365
	**48–95 years**	105 (48.4)	255 (202–325)	
GCS**, **n (%)**				
	**15**	192 (88.5)	244 (147–312)	<0.001
	**13 or 14**	25 (11.5)	327 (254–408)	
ISS score** **(range)**				
	**Lower (0–1)**	128 (59.0)	232 (121–314)	0.007
	**Higher (2–13)**	89 (41.0)	261 (210–350)	
**Laboratory tests**				
	**No**	159 (73.3)	225 (124–294)	<0.001
	**Yes**	58 (25.7)	347 (271–411)	
**Treatment of wounds**				
	**No**	138 (63.6)	227 (130–310)	<0.001
	**Yes**	79 (36.4)	298 (219–380)	
**Number of CT scans**				
	**Lower (0–2)**	196 (90.3)	244 (151–312)	<0.001
	**Higher (3–4)**	21 (9.7)	394 (295–430)	
**Number of X-rays**				
	**Lower (0–1)**	136 (62.7)	233 (146–303)	0.005
	**Higher (2–7)**	81 (37.3)	288 (199 357)	

*Analyzed using the Mann-Whitney U-tests.

**GCS, Glasgow Coma Scale; IQR, interquartile range

**Fig 2:**
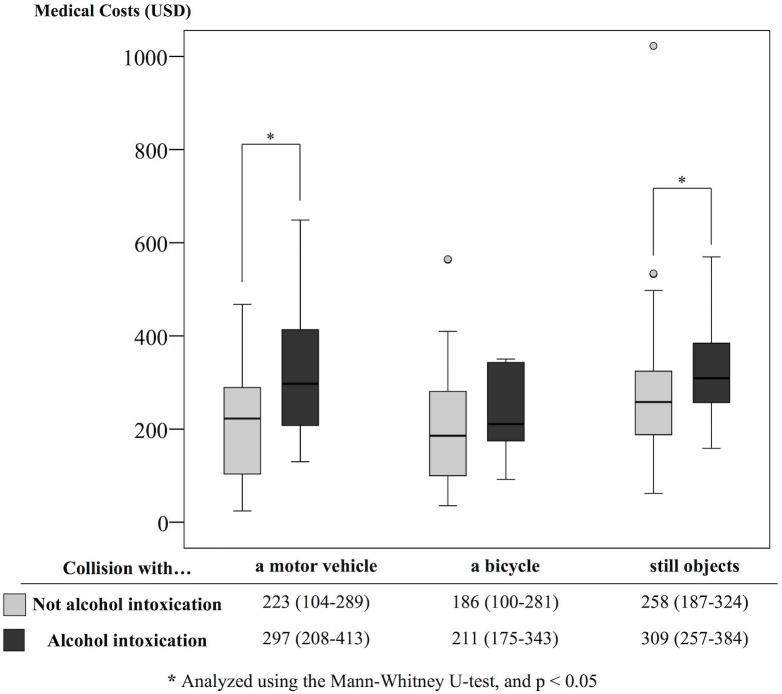
Boxplot of medical costs by type of accident. Median (IQR) of medical costs are shown by intoxication status of the patient.

Fig 2 shows the medical costs by type of accidents. Medical costs do not significantly differ by accident type overall and even when stratified by intoxication status. When comparing medical costs between intoxicated and not intoxicated patients, a statistically significant association was found for collision with a motor vehicle (297 [208–413] vs. 223 [104–289], *p* = 0.027) and collision with still objects (309 [257–384] vs. 258 [187–324], *p* = 0.002); a similar tendency was observed for collision with a bicycle as well.

Multiple linear regression analysis (Table 3) showed that alcohol intoxication was independently associated with higher medical costs (beta coefficient = 60.08, 95% CI = 18.57–101.60, *p* = 0.005) after adjusting for sex, age, collision with a motor vehicle, GCS, and ISS (Model 1). Additional adjustment (Model 2) including clinical tests and treatment (i.e. laboratory tests, treating wounds, number of X-rays, and number of CT scans) attenuated the effect of alcohol intoxication on medical cost, but was still associated with a statistically significant association (beta coefficient = 34.05, 95% CI = 3.35–64.75, *p* = 0.030). Adjusted R-square value determined using multiple linear regression was 0.55 indicating reasonable fit of the statistical model to the data.

**Table 3:** Multiple linear regression analyses for medical costs associated with alcohol intoxication among bicyclists visiting the ED for minor injuries.

	Model 1*: Adjusted R-square = 0.13			Model 2**: Adjusted R-square = 0.55		
	β coefficient	95%CI***	*p*-value	β coefficient	95%CI***	*p*-value
**Intercept**	279.09			105.96		
**Alcohol intoxicated**	60.08	18.57–101.60	0.005	34.05	3.35–64.75	0.030
**Male sex**	-30.33	-65.31–4.64	0.089	-17.83	-43.30–7.65	0.169
**Age**	-0.21	-1.18–0.77	0.676	-0.51	-1.21–0.20	0.158
**Collision with a motor vehicle**	-30.60	-67.52–6.32	0.104	-22.83	-49.77–4.10	0.096
GCS*** **of 15**	-32.58	-90.00–24.84	0.265	31.62	-10.99–74.24	0.145
ISS***	11.80	1.50–22.11	0.025	0.50	-7.13–8.12	0.898
**Laboratory tests**	-	-	-	79.99	50.05–109.93	< 0.001
**Treatment of wounds**	-	-	-	46.46	20.03–72.89	0.001
**Number of X-rays**	-	-	-	31.02	21.17–40.88	< 0.001
**Number of CT scans**	-	-	-	56.60	44.01–69.19	< 0.001

* Adjusted for sex, age, collision with motor vehicle, GCS, and ISS.

**Adjusted for sex, age, collision with motor vehicle, GCS, ISS, laboratory tests, treatment of wounds, number of X-rays, and number of CT scans.

***GCS, Glasgow Coma Scale; ISS, injury severity score; CI, confidence interval.

## Discussion

We show here that alcohol intoxication was independently associated with increased medical costs of minor injuries caused by bicycle-related crashes. Medical costs in Japan are defined by the type of testing and treatment that are conducted in the ED. Because alcohol intoxication complicates diagnosis and decisions for providing the most appropriate treatment, it is likely that intoxicated patients more often require additional medical care even for minor injuries. Further, the use of a clinical prediction rule, such as the New Orleans Criteria for minor head injury[11] and National Emergency X-Radiography Utilization Study (NEXUS)[12] for minor cervical spine injury, to inform decision-making for imaging testing is not appropriate for intoxicated patients. Our results showing that the rate of head and cervical CT scans who consumed alcohol are higher than those who did not consume alcohol support this hypothesis.

Compared to intoxicated patients, those who were not intoxicated were more frequently involved in a collision with a motor vehicle. In contrast, intoxicated patients were more frequently involved in self-accidents. This observation may reflect a tendency for non-intoxicated patients to be involved in less severe self-accidents and/or have a greater ability to manage self-accidents without need for an ambulance; whereas, collision with a motor vehicle usually warranted calling an ambulance. We observed that the type of accident was not significantly associated with medical costs. Thus, we would not expect medical costs to significantly change even if the proportion of types of accidents change.

Our findings support the hypothesis that medical costs associated with treating minor injuries suffered by bicyclists who are intoxicated are higher than those who did are not. Understandably, our findings also indicate that laboratory tests, treating wounds, number of X-rays, and CT scans are independently associated with an increase in medical costs. Interestingly, a significant effect of alcohol intoxication remained even after adjustment for the obvious major factors believed to be contributing to the higher medical costs. Overall, not only do our finding demonstrate an important influence of intoxication, but also suggests that additional factors related to alcohol intoxication, other than those evaluated, may effect medical costs as well.

A previous study has shown that medical costs increase as the severity of trauma gets worse[13], and several studies have shown that bicycle-related injuries suffered by intoxicated riders are significantly associated with increases in severity of trauma and hospitalization or death[14, 15]. Similarly, intoxicated patients incur increased costs [8, 9]. To the best of our knowledge, this is the first study to evaluate the association between alcohol intoxication and an increase in medical costs for bicycle-related outpatient minor injuries. We conclude that medical costs can be reduced by minimizing bicycle riding of intoxicated individuals. The results should encourage the proper authorities to take further steps to prohibit bicycling under the influence of alcohol as a way to prevent injuries and reduce medical costs.

The present study has some limitations. First, this was a single-center study, which may have affected the generalizability of findings. However, our hospital accepts patients from rural and urban areas regardless of the severity of their injuries. Therefore, at a minimum, the findings can be generalized to an average population in Japan. Because the level of alcohol intoxication was judged according to subjective criteria, the results may have been affected by this misclassification which can be expected to be non-differential in nature leading to a potential under-estimate of the magnitude of effect. In Japan, few hospitals measure blood alcohol concentration. Additionally, it has been shown that a blood alcohol concentration that would impair driving and be punishable in the US does not always produce reliable subjective signs of alcohol intoxication[16]. Several studies have also shown that physical signs of alcohol intoxication do not correlate with blood alcohol concentration[16, 17]; thus, riding a bicycle under the influence of alcohol is judged subjectively by Japanese law[5]. Further, previous reports have shown that the relationship between increased medical costs and bicycle-related injuries under the influence of alcohol[9], and the risk of bicycle-related crashes caused by alcohol intoxication is similar when measured using a blood test or a self-report[18]. Finally, due to the unexpected observation of a residual effect of alcohol intoxication on medical costs even after adjustment, collection of a larger breath of covariate data would further aid our understanding of the factors contributing to the mechanisms associated with higher medical costs in alcohol intoxicated patients.

We conclude from our results that higher medical costs are associated with alcohol intoxication among bicyclists visiting the ED for minor injuries. This association was independent of the effect of injury severity and other related factors. It is still very common for people to ride a bicycle under the influence of alcohol despite the law that prohibits it. To prevent crashes and reduce healthcare costs, a greater emphasis should be placed on enforcement of laws that prohibit people from bicycling when they are under the influence of alcohol.
